# Overall survival of stage IV non-small cell lung cancer patients treated with *Viscum album *L. in addition to chemotherapy, a real-world observational multicenter analysis

**DOI:** 10.1371/journal.pone.0203058

**Published:** 2018-08-27

**Authors:** Friedemann Schad, Anja Thronicke, Megan L. Steele, Antje Merkle, Burkhard Matthes, Christian Grah, Harald Matthes

**Affiliations:** 1 Research Institute Havelhöhe, Hospital Havelhöhe, Berlin, Germany; 2 Interdisciplinary Oncology and Palliative Care, Hospital Havelhöhe, Berlin, Germany; 3 Institute of Health and Biomedical Innovation, Queensland University of Technology, Brisbane, QLD, Australia; 4 Lung Cancer Center and Department of Pneumology, Hospital Havelhöhe, Berlin, Germany; 5 Medical Clinic for Gastroenterology, Infectiology and Rheumatology CBF and Institute of Social Medicine, Epidemiology and Health Economics CCM, Charité University Medicine Berlin, Berlin, Germany; Peking University People's Hospital, CHINA

## Abstract

**Background:**

Stage IV non-small cell lung cancer (NSCLC) is associated with a five-year survival rate of around 1%. Treatment with Viscum album L. (VA) extracts has been shown to reduce chemotherapy (CTx)-related adverse events, decrease CTx dose reductions and improve quality of life in a number of cancers. Recent data suggest a beneficial effect of add-on treatment with *Viscum album L*. (VA, European mistletoe) on survival in cancer patients. The objective of this study was to evaluate the effect of VA in addition to chemotherapy on survival in stage IV NSCLC patients.

**Methods:**

The observational study was conducted using data from the Network Oncology clinical registry which is an accredited conjoint clinical registry of German oncological hospitals, practitioners and out-patient centers.Patients were included if they had stage IV NSCLC at diagnosis, lived at least for four weeks post-diagnosis and received chemotherapeutic treatment. Patients with EGFR mutations as well as patients receiving tyrosine kinase inhibitors or immune checkpoint inhibitors were not included. Overall survival and impact on hazard in patients with chemotherapy (CTx) to patients receiving CTx plus VA were compared. To identify factors associated with survival and to address potential sources of bias a multivariate analyses using Cox proportional hazard model was performed.

**Results:**

The median age of the population was 64.1 years with 55.7% male patients. The highest proportion of patients had adenocarcinoma (72.2%) and most of the patients were current or past smokers (70.9%). Of 158 stage IV NSCLC patients, 108 received CTx only and 50 additional VA. Median survival was 17.0 months in the CTx plus VA group (95%CI: 11.0–40.0) and was 8.0 months (95%CI: 7.0–11.0) in the CTx only group (χ^2^ = 7.2, p = .007). Overall survival was significantly prolonged in the VA group (HR 0.44, 95%CI: 0.26–0.74, p = .002). One-year and three-year overall survival rates were greater with CTx plus VA compared to CTX alone (1y: 60.2% vs. 35.5%; 3y: 25.7% vs. 14.2%).

**Conclusion:**

Our findings suggest that concomitant VA is positively associated with survival in stage IV NSCLC patients treated with standard CTx. These findings complement pre-existing knowldedge of add-on VA’s clinical impact, however, results should be interpreted with caution in light of the study’s observational character.

## Introduction

According to the Global Health Observatory data of the World Health Organization, cancer of the trachea, bronchus and lung account for 1.7 million deaths worldwide which makes them being the world’s top five cause of deaths [1]. Accounting for 25.9% of all cancer-related deaths in 2017, lung & bronchus cancer ranks first position, followed by breast, colon & rectum, prostate and pancreatic cancer [2]. Almost 85% of U.S. lung cancers are non-small cell lung carcinoma (NSCLC) [3]. Over one half of primary NSCLC patients are already diagnosed with stage IV lung cancer. The median overall survival (OS) of these patients ranges between 7.0 and 12.2 months depending on treatment, histology type and other associated factors [4–6]. As stage IV NSCLC is one of the most devastating diagnoses of lung cancer, worldwide great effort is done in the search for new treatment solutions, i.e. reflected by the vast clinical research on CTX-combinations in the past and accelerated approval of new immuno-oncological treatment in the U.S. and Europe in recent years [7, 8].

Even though newer histology and molecular pattern-based treatments including immune checkpoint inhibitors (ICI) are preferred as they are beneficial for patients with specific molecular subtypes [9, 10], chemotherapeutic combinations still play a major role in first-line treatment. Generally, platinum-based CTx is the first-line therapy in patients with advanced NSCLC without any targetable mutations. For metastatic non-squamous NSCLC (showing less than 50% PD-L1 expression) bevacizumab revealed a survival benefit in combination with carboplatin and paclitaxel as first-line treatment [11]. For first-line treatment of patients with metastatic NSCLC whose tumors have at least 50% PD-L1 expression with no EGFR-, ALK positive or other activating tumor mutations, the immune checkpoint inhibitor pembrolizumab [12] has been approved. A high proportion of patients experience disease progression during or after gold-standard CTx regimen. Despite reported efficacy and effectiveness, the tolerability of current modern oncological treatment with respect to health-related quality of life (HRQL) and palliative care remains an important issue [13–19]. Thus, the quest for an effective treatment regimen with a sound safety profile in this field continues.

VA is applied in integrative oncology concepts concomitantly to CTx to improve HRQL [20–25]. Even though the evidence of VA’s impact on survival is discussed controversially [25–27] and a Cochrane review in 2008 summarizes that “there was no consistent effect of mistletoe extracts” on clinical outcome [25], its potential beneficial effect on survival of cancer patients is accumulating [21, 25, 28–32]. The objective of the present observational study was to evaluate the effect of additional VA treatment on the survival of stage IV NSCLC patients treated with standard CTx.

## Materials and methods

### Study design and patients

A non-controlled, non-randomized observational multicenter cohort study was conducted revealing,real-world, data (RWD) [33] by analysing patient registry data (Network Oncology, NO). The NO is a conjoint clinical register of hospitals, practitioners and out-patient centers [34] of which three study centers participated. Patients were included who were 18 years or older, who gave written consent, with a histologically proven primary diagnosis of stage IV non-small cell lung carcinoma seen between February 2010 and June 2016, receiving chemotherapeutic treatment surviving more than 28 days. Patients were not included if they did not give written consent, received any targeted therapies including monoclonal antibodies, tyrosine kinase inhibitors or any ICI, or when death date or last contact date were not available. Follow-up was performed routinely six months after first diagnosis and annually during the next years. Loss to follow-up was defined as no follow-up visits.

### Ethics approval and consent to participate

This study is an observational cohort study of the Network Oncology (NO) which has been approved by the ethical committee of the Medical Association Berlin (Berlin—Ethik-Kommission der Ärztekammer Berlin). The reference number is Eth-27/10. Written informed consent has been obtained from all patients prior study enrolment. The study complies with the principles laid down in the Declaration of Helsinki.

### Data collection

Structured Query Language inquiries on records of patients were run for lung cancer patients (International Classification of Diseases code: C34) using the clinical database NO. For queried patients demographic data and hospital-related data (diagnosis, histology, pre-treatment and treatment) were retrieved from the NO. In addition, recorded TNM stages or documented metastases were queried with their according date and translated into Union for International Cancer Control (UICC) stages according to the 7^th^ edition of TNM Classification of Malignant Tumors [35]. UICC stage at first diagnosis was defined as the earliest recorded stage within a month of the diagnosis date. Furthermore, chemotherapeutic applications were queried with their according date. Surgical interventions were coded according to the German procedure classification 2013 [DIMDI, http://www.dimdi.de/static/en/klassi/ops/index.htm]. Application of VA extracts in the context of an integrative oncological setting was retrieved with start and end dates, application type and the pharmaceutical used. VA therapy was defined as lasting more than four weeks. Best objective responses were assessed on patients with measurable disease. Tumor response was assessed according to revised RECIST guidelines, version 1.1 for solid tumors [36]. Progression-free survival was defined as time from date of index date to documented disease progression (according to RECIST) or death from any cause [37]. Patients who were alive but had not progressed at the time of the analysis were censored.

### Classification of groups

Included NSCLC patients were classified into the histological subgroups non-squamous cell carcinoma, squamous cell carcinoma or large cell carcinoma. We then classified patients to one of the two groups: a) CTx group—patients received only CTx and no VA therapy and b) CTx + VA group–patients that received concomitant VA therapy. CTx only or CTx+ VA were applied as per routine clinical care. Non-randomized allocation to the treatment groups was performed by the physician after elaborate information and patient’s decision on treatment options. Applied VA preparations included Abnobaviscum, Helixor and Iscador VA extracts. VA therapy was applied subcutaneously according to SmPC [38–40]. Off-label intravenous application was performed in individual cases.

### Determination of sample size

For a two-sided sample size test assuming a power of 80% and 5% level of significance with an allocation scheme of 0.3 (CTx/VA) to 0.7 (CTx) and a relative hazard of 0.43 [32], a total of 156 patients (47 patients in the CTx/VA and 109 patients in the CTx group) would be needed to confirm a statistically significant treatment effect according to Schoenfeld et al. [41]

### Endpoints

The objective of this study was to evaluate the effect of VA in addition to CTx on survival in stage IV NSCLC patients. The primary outcome of the study was the evaluation of OS and to test the hypothesis that stage IV NSCLC patients receiving additional VA to CTx have a longer OS than patients receiving CTx only. The secondary outcome was the assessment of factors for their association with the hazard of dying.

### Statistical analysis

The start date for survival analysis was the first date of available histology (index date) which was +/- 28 days of date of first diagnosis of stage IV lung cancer. Patient survival was calculated from index date until the patient's last record, which was either the date of death, or the last documentation of personal contact, interdisciplinary tumor board or follow-up (for follow-up measures please see,study design and patients,). A year lasted 365.25 days and a month was 365.25/12 days. Kaplan Meier survival was calculated for both groups.

To analyse how different factors influence the hazard on patient survival and to reduce potential confounding bias we employed multivariate stratified Cox proportional hazard model adjusting for demographic, histological and treatment variables as well as smoker-status. Potential confounders that were addressed were age, gender, BMI, smoking status, and oncological treatment. Prior this analysis verification analyses were performed whether or not proportional hazard assumptions were met. All analyses were conducted using the software R, version 3.3.0–2016-05-03, R-Studio version 0.99.896, a language and environment for statistical computing [42]. Continuous variables were described as median with interquartile range (IQR); categorical variables were summarised as absolute and relative frequencies. Data distributions were inspected graphically using box plots and histograms and were arithmetically examined for skewness. Patients with missing data were not included. For both groups, baseline characteristics and treatment regimens were compared using the unpaired Student’s t-test for independent samples. For comparison of categorial variables chisquare analysis was performed. All tests were performed two-sided. P-values < .05 were considered significant.

For survival analysis including Kaplan-Meier curves and right-censored time-to-event analyses as well as univariate and multivariate Cox proportional hazard models the R-package ‘survival’ was applied, version 2.41–3 published by Terry M. Therneau and Thomas Lumley on 2017-04-04; https://CRAN.R-project.org/package=survival. For the implementation of nonparametric estimators for censored event history (survival) analysis the package ‘prodlim’ was applied, version 1.6.1 published by Thomas A. Gerds 2017-03-06 (https://CRAN.R-project.org/package=prodlim). To draw survival curves the package ‘survminer’ was used, version 0.4.0 by Alboukadel Kassambara, Marcin Kosinski, Przemyslaw Biecek published 2017-06-07 (https://CRAN.R-project.org/package=survminer).

## Results

### Patients

**Fig 1:**
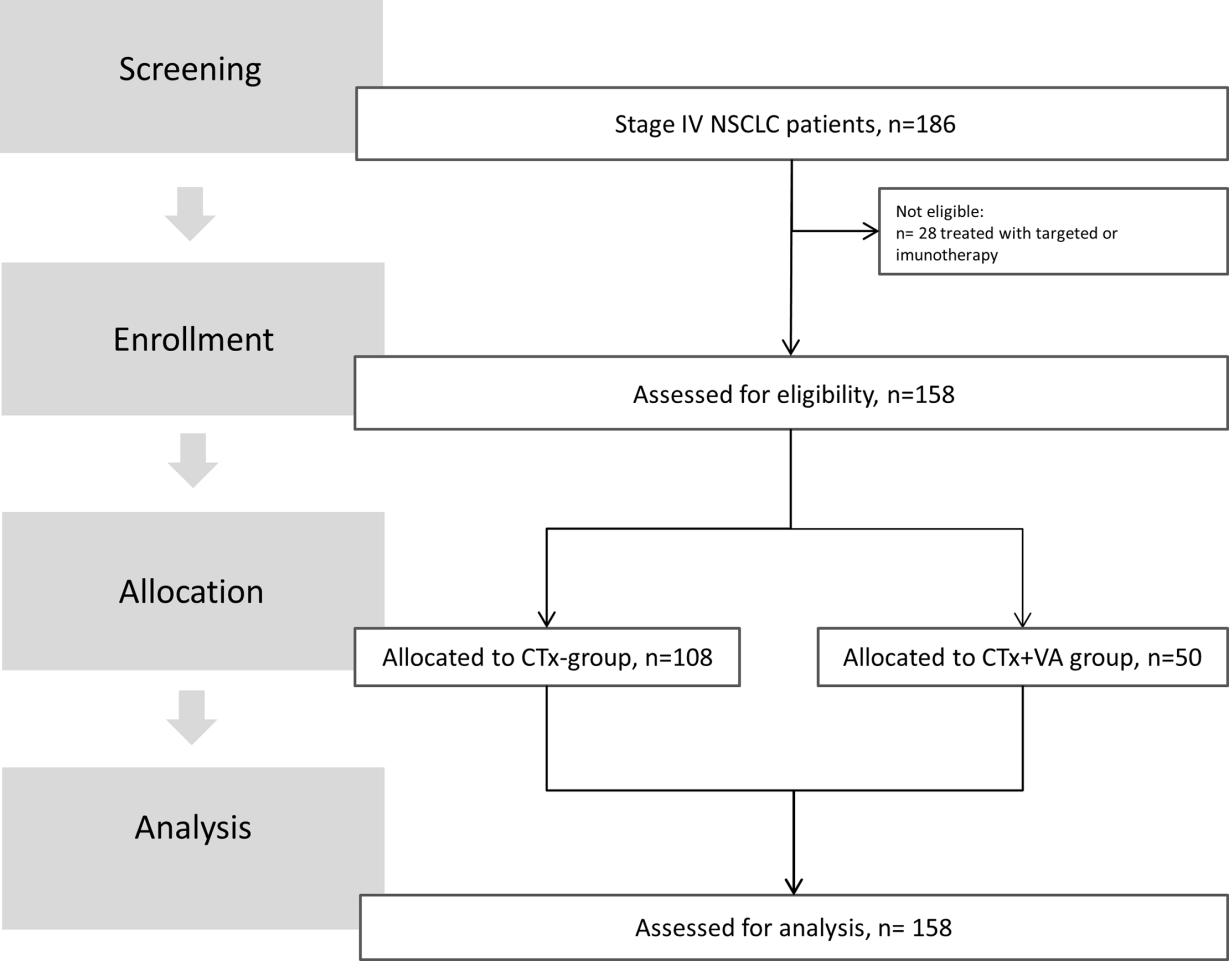
Flow chart of the study population. NO, network oncology; CTx, Chemotherapy; VA, Viscum album L., mistletoe.

158 stage IV NSCLC cancer patients that were diagnosed between February 2010 and June 2016 (follow-up total: 2280 days; average 228.6 days) in the NO revealed complete histological data, received CTx, and showed survival of greater 28 days after index date rendering eligibility of these patients for subsequent survival analysis (see study flow chart, Fig 1). Non-eligibility for analysis was characterized by receival of targeted or ICI therapy (n = 28).

Table 1 shows the main characteristics of analyzed patients at baseline. No significant differences between groups with regards to demographic characteristics, tumor histology subtypes, smoker status, cancer-directed surgery, radiation were seen. 108 patients received only CTx and 50 patients received VA in addition to CTx. Mean age of the total cohort was 64.1 years with no significant differences between both groups. The sex ratio (male/female) was 1.26 in the total cohort. The percentage of current/past smokers and of never-smokers was slightly higher in the CTx group (p = .08).

**Table 1:** Characteristics of patients with stage IV non-small cell lung cancer.

	All patients (n = 158)		CTx (n = 108)		CTx + VA (n = 50)		
	N	%	N	%	N	%	P-value^1^^)^
Total number of patients	158	100	108	68.4	50	31.6	
Age at diagnosis, mean (SD), years	64.1	(10.4)	63.9	(10.6)	64.5	(10.1)	0.73
Gender							
Female	70	44.3	48	44.4	22	44.0	
Male	88	55.7	60	55.6	28	56.0	1.00
Year							
2010–2013	98	62.0	62	57.4	36	72.0	0.11
2014–2016	60	38.0	46	42.6	14	28.0	
Body mass index							
<25	76	48.1	50	46.3	26	52.0	
25–29.9	41	25.9	27	25.0	14	28.0	
30+	12	7.6	10	9.3	2	4.0	
Unknown	29	18.4	21	19.4	8	16.0	0.61
Histology							
Non-squamous carcinoma	114	72.2	76	70.4	38	76.0	
Squamous cell carcinoma	31	19.6	25	23.1	6	12.0	
Large cell carcinoma	13	8.2	7	6.5	6	12.0	0.17
Smoker							
Current/Past	112	70.9	81	75.0	31	62.0	
Never	15	9.5	11	10.2	4	8.0	
Unknown	31	19.6	16	14.8	15	30.0	0.08
Cancer-directed surgery							
No	111	86.7	74	88.1	37	84.1	
Yes	17	13.3	10	11.9	7	15.9	0.72
Radiation therapy							
No	85	53.8	61	56.5	24	48.0	
Yes	73	46.2	47	43.5	26	52.0	0.41

Characteristics of patients with stage IV non-small cell lung cancer, percentages of sub-characteristics may not add up to 100% due to rounding procedures, VA = Viscum album L., SD = standard deviation

^1)^ chisquare analysis for categorial variables; Student’s t-test for age distribution

Of the total analysed cohort 72.2% (n = 114) had non-squamous, 19.6% (n = 31) squamous cell carcinoma and 8.2% (n = 13) large cell carcinoma. The percentage of patients diagnosed with squamous cell carcinoma was two times higher in the CTx compared to the combinational CTx + VA group and the percentage of patients with large cell carcinoma was two times higher in the combinational CTx + VA group compared to the CTx group; differences in the proportions of histology classes between both groups were not significant.

### Oncological treatment

As to first-line CTx treatment, platinum-compounds were received by 116 (73.4%) of all patients, mostly in combination with gemcitabine, pemetrexed, vinorelbine or etoposid, see Table 2. Gemcitabine was applied to 11 (7%) patients either as monotherapy or in combination with platinum compounds or vinorelbine at some point. Docetaxel and paclitaxel were the most frequent taxanes (22 patients, 13.9%) and mainly given as monotherapy or in combination with platinum compounds. 24 (15.2%) patients received bisphosphonates, one patient received hormonal therapy, namely bicalutamide, data not shown. As to first-line CTx treatment no significant differences were seen between both groups, data not shown. For those patients for whom second-line treatment data was retrieved, no significant differences between both groups were detected, data not shown.

**Table 2:** Composition of CTx regimen.

	N (%)
CTx	158 (100)
platinum compounds	116 (73.4)
vinorelbine	42 (26.6)
pemetrexed	67 (42.4)
taxanes (docetaxel, paclitaxel)	22 (13.9)
etoposide	13 (8.2)
gemcitabine	11 (7.0)

First-line chemotherapy applied to patients with stage IV NSCLC (n = 158). Numbers in rows and columns do not necessarily add to one hundred percent as patients may have received various combinations of preparations. N, number; %, percent; CTx, chemotherapy.

In addition to CTx, 50 patients (31.7%) received extracts of VA, see Table 3. The most frequent type of application for mistletoe agents was subcutanous injection in 44 patients (88.0% of all VA patients), followed by off-label intravenous or intratumoral injections in 40 (80%) and 3 (6.0%) patients, respectively. In general, Abnobavisucm extracts were the mistletoe remedies most often prescribed (n = 42), especially for subcutaneous application (n = 35), followed by Helixor remedies, mainly used for intravenous application (n = 31) and Iscador preparations (n = 12).

**Table 3:** Number of stage IV NSCLC patients receiving additional VA to standard CTx.

	Total	Abnobavisum preparations	Iscador preparations	Helixor preparations
Total number of patients, n (%)	50 (100)	42 (100)	12 (100)	31 (100)
Subcutaneous application, n (%)	44 (88.0)	35 (83.3)	12 (100)	1 (3.2)
Intravenous application, n (%)	40 (80.0)	13 (31.0)	2 (16.7)	31 (100)
Intratumoural application, n (%)	3 (6.0)	3 (7.1)	0	0

Characteristics of VA therapy and application type applied additionally to CTx (n = 50). Numbers in rows and columns do not necessarily add to one hundred percent as patients may have received various combinations of preparations. n, number; %, percent, VA, Viscum album L. (mistletoe)

### Outcomes

One hundred and fifty-eight patients were included in the overall survival (OS) analysis from which 86% (n = 136) died during total observational time with 24.1% (n = 38) in the CTx + VA group and 62% (n = 98) in the CTx only group.

**Fig 2:**
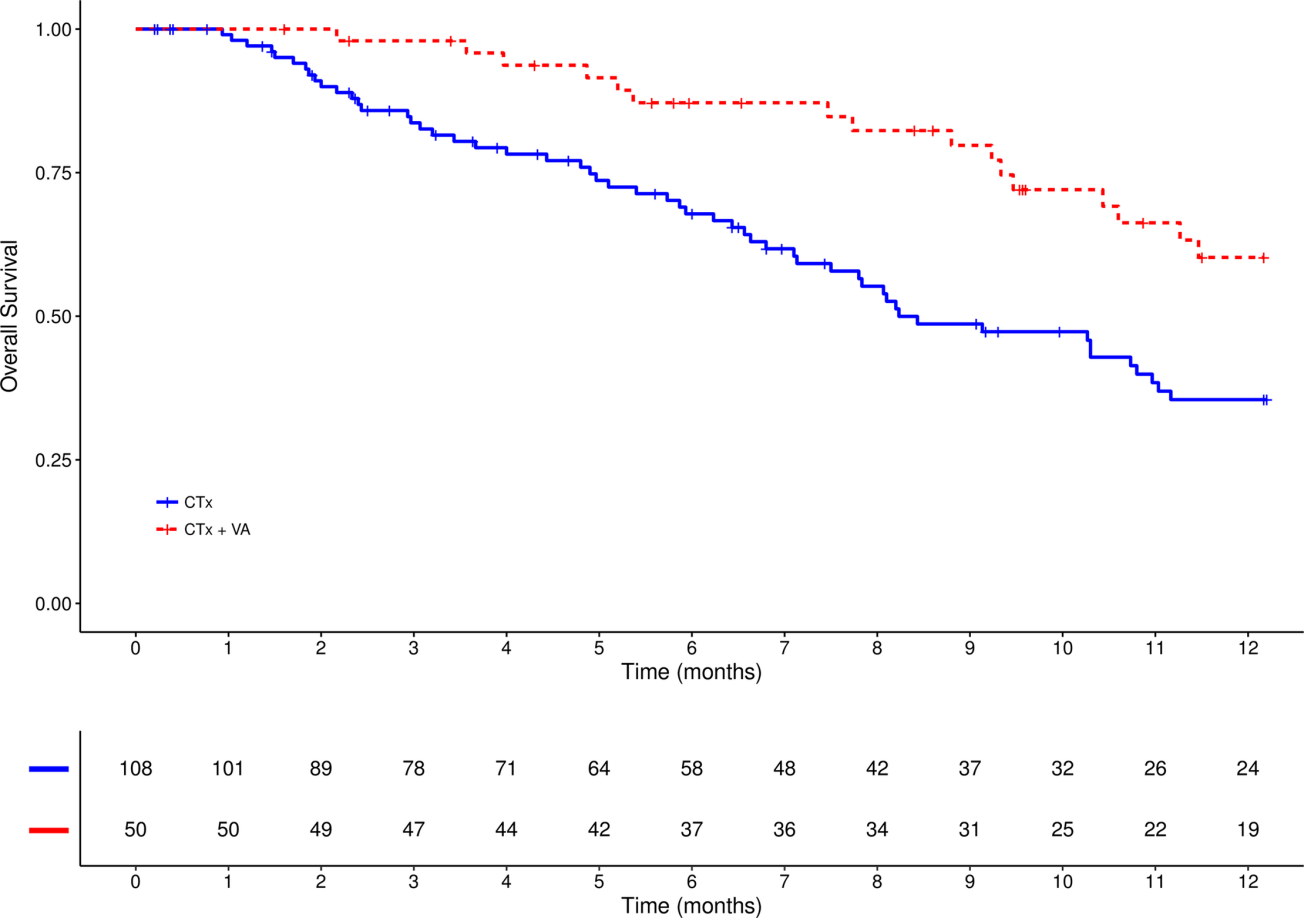
One-year survival. Kaplan–Meier survival curves displaying one-year survival in stage IV NSCLC patients treated either with CTx alone or with combinational CTx plus VA, n = 158; CTx, chemotherapy; VA, Viscum album L.

**Fig 3:**
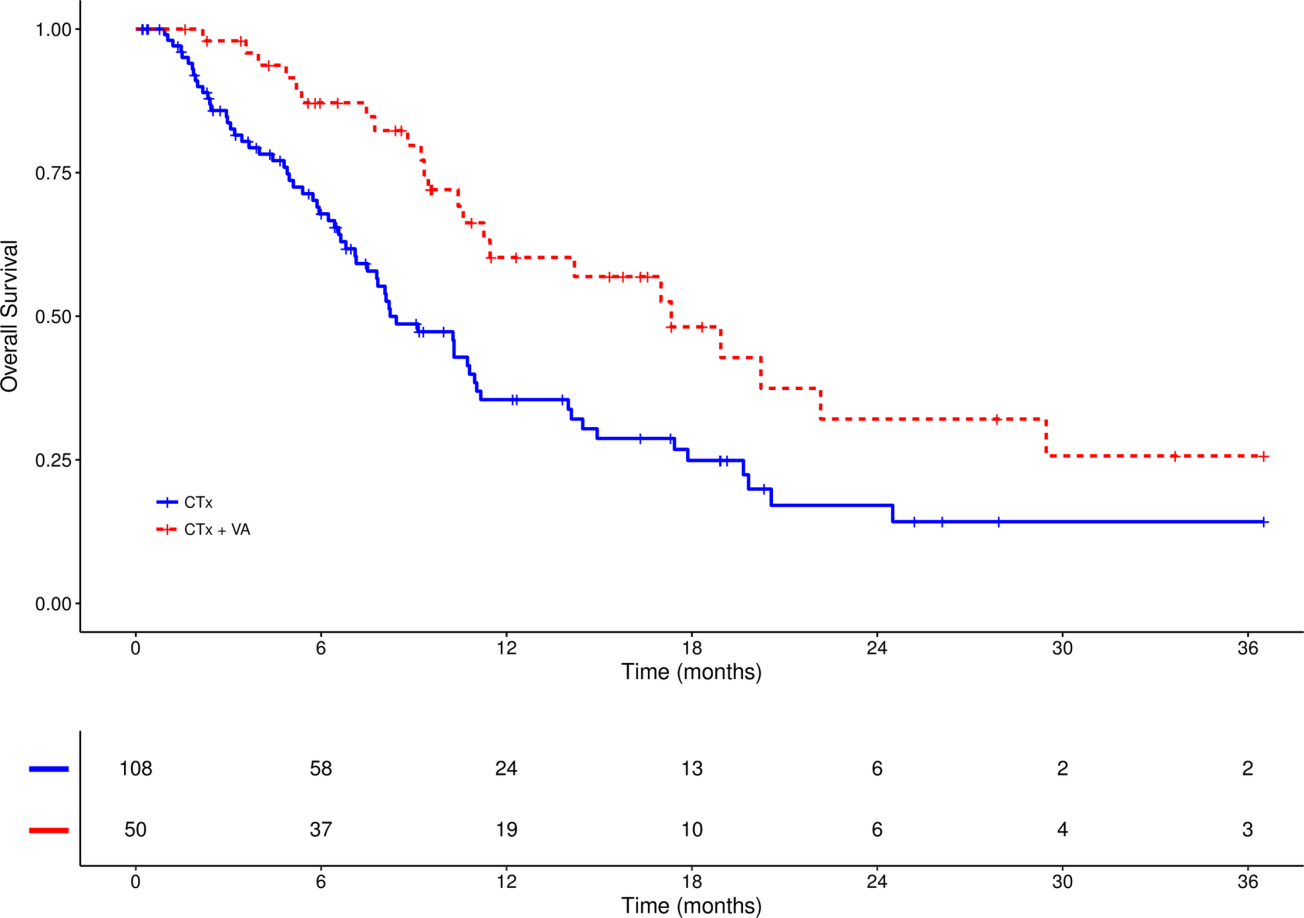
Three-year survival. Kaplan–Meier survival curves displaying 3-year survival in stage IV NSCLC patients treated either with CTx alone or with combinational CTx plus VA, n = 158; CTx, chemotherapy; VA, Viscum album L.

As to overall survival a survival benefit was seen for the combinational treatment (CTx + VA) compared to CTx only, see Figs 2 and 3. The median OS was 17.0 months in the CTx plus VA group (95%CI: 11.0–40.0) and was 8.0 months (95%CI: 7.0–11.0) in the CTx only group, see Table 4. This difference was statistically significant (χ^2^ = 7.2, p = .007). One-year OS rates were 35.5% for the CTx-group and 60.2% for patients who received additional VA; three-year OS rates were 14.2% for the Ctx-group and 25.7% for the combinational CTx plus VA group.

**Table 4:** Median overall survival of patients with stage IV NSCLC.

	N	Events	Median [months]	CI [months]
CTx	108	64	8	[7–11]
CTx & VA	50	25	17	[11–40]
Log rank test Χ^2^ = 7.2 on 1 degrees of freedom, p = 0.007				

Median overall survival, n = 158. CTx, chemotherapy, VA = Viscum album L., SD = standard deviation

**Fig 4:**
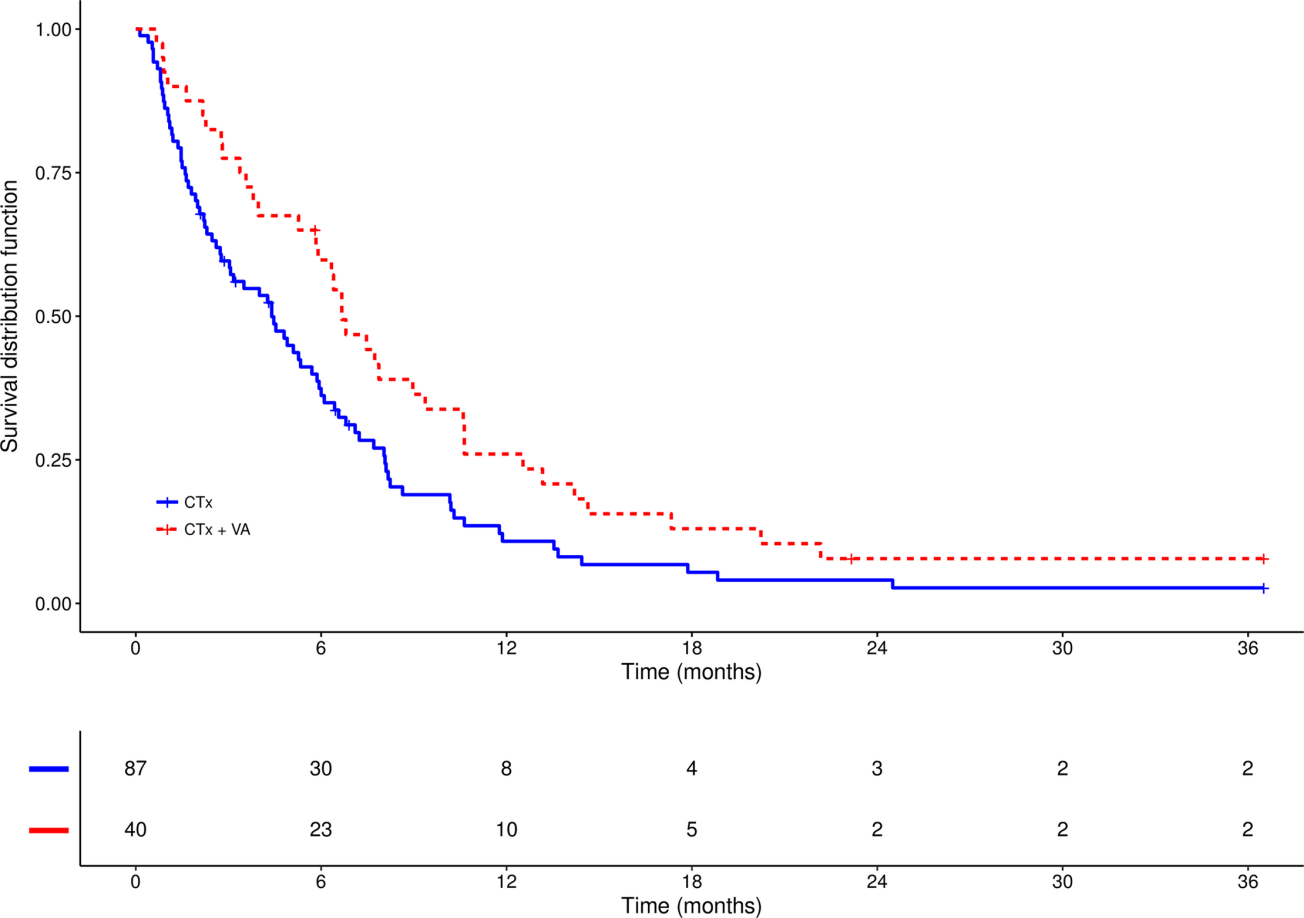
Kaplan-Meier curves of progression-free survival (PFS) for the both treatment cohorts CTx and CTx + VA. Median PFS CTx: 4.4 months (95%CI: 2.7–5.9) vs. median PFS CTx+VA: 6.7 months (95%CI: 4.0–9.0), χ2 = 3.4, p = 0.06.

Progression-free survival (PFS) analysis was performed in a subgroup of 126 patients (79.7%) and revealed a tendency towards significance (χ2 = 3.4, p = 0.063), for a prolonged median PFS in the CTx + VA group (6.7 months; 95%CI: 4.0–9.0) compared to the CTx group (4.4 months; 95%CI: 2.7–5.9) 6.4% see Fig 4.

Response rates have been analysed in a subgroup (n = 126, 79.7%) for which data were retrievable, see Table 5. Both groups showed similar low rates of complete response. In a significantly higher proportion of patients of the CTx group the disease progressed (87.4%) compared to 66.7% in the CTx + VA group (p = 0.01). A highly significant difference (p = 0.008) was observed for partial responders between both groups with a higher proportion in the CTx + VA group (30.8%) compared to the CTx only group (10.3%).

**Table 5:** Tumor response after treatment with CTx with or without VA therapy (subgroup, n = 126).

Disease response	CTx (n = 87)	CTx + VA (n = 39)	p-Value
Complete response, n (%)	1 (1.1)	1 (2.6)	.53
Partial response, n (%)	9 (10.3)	12 (30.8)	.008
Stable disease, n (%)	1 (1.1)	0 (0)	1
Progressive disease, n (%)	76 (87.4)	26 (66.7)	.013

Additional VA therapy compared to no VA therapy significantly reduced hazard by 48% as shown by univariate Cox proportional hazard model (HR: 0.52, 95% CI = 0.33–0.83, p = .007) (data not shown). Hazard was significantly reduced by 52% when the duration of add-on VA therapy was prolonged to ≥16 weeks (HR: 0.48, 95%CI: 0.28–0.83, p = .007). Highly statistically significant reduction of hazard of death remained after adjusted multivariate stratified Cox proportional hazard analysis revealing a hazard reduction of 56% (adjusted hazard ration–aHR: 0.44, 95%CI = 0.26–0.74, p = .002), see Table 6. Nine variables including age, gender, concomitant VA therapy, body mass index, histology, smoker status, cancer-directed surgery and radiation were adjusted. Cancer-directed surgery and radiation therapy showed no significant effect on hazard, while the direction of impact on hazard was indicated to be negative. Male gender compared to female gender significantly increased hazard of death by factor 1.7 (aHR: 1.65, 95%CI: 1.04–2.62, p = .034). An unknown status of BMI was statistically associated with an increased risk (aHR: 2.32, 95%CI: 1.20–4.49, p = .01). Other factors showing an association with increased adjusted hazard of death were histology subtype large cell carcinoma (aHR: 3.74, 95%CI: 1.78–7.85, p = .0005) compared to subtype adenocarcinoma.

**Table 6:** Multivariate regression analysis of factors associated with hazard of death in stage IV NSCLC patients treated with CTx.

	aHR	(95% CI)
Total number of patients n = 158		
Age, median (IQR), years	1.00	(0.98–1.03)
Gender		
Female	1	Reference
Male	1.65*	(1.04–2.62)
Add-on VA therapy		
No	1	Reference
Yes	0.44**	(0.26–0.74)
Body mass index		
<25	1	Reference
25–29.9	0.86	(0.51–1.50)
30+	0.51	(0.19–1.35)
Unknown	2.32*	(1.20–4.49)
Histology		
Non-squamous carcinoma	1	Reference
Squamous cell carcinoma	1.45	(0.84–2.48)
Large cell carcinoma	3.74***	(1.78–7.85)
Smoker		
Never	1	Reference
Current/Past	1.41	(0.62–3.22)
Unknown	0.76	(0.27–2.12)
Cancer-directed surgery		
No	1	Reference
yes	0.78	(0.50–1.24)
Radiation		
No	1	Reference
yes	0.72	(0.45–1.15)

Multivariate regression analysis, adjusted hazard ratio based on Cox proportional hazard model, model for each group adjusted for demographic variables, VA treatment, smoking status, histology class, body mass index, receipt of cancer-directed surgery and radiation. Stratified variables are not shown; aHR, adjusted hazard ratio; CI, confidence interval.

*P ≤0.05

** P ≤0.005

*** P ≤0.001.

Except age as being a continuous variable all other explanatory variables were of categorical nature.

## Discussion

The results of the present study reveal a significant survival benefit for metastasized NSCLC patients that received CTx in combination with VA therapy compared to patients being treated with CTx alone. Adjusted multivariate stratified Cox proportional hazard analysis showed that concomitant VA therapy reduced hazard of death by 56% in stage IV NSCLC patients treated with CTx compared to CTx only treatment. Our findings are in line with a meta-analysis in 2012 which stated a general reduced hazard after additional VA therapy (overall HR 0.59, 95% CI: 0.50–0.70) [30]. In line with this, a survival benefit due to subcutaneous VA plus best supportive care compared to best supported care only (HR 0.49, 95% CI 0.36–0.65) was shown by Tröger and collegues in advanced or metastatic pancreatic cancer patients (n = 220) [32]. In this study survival was improved by 2.1 months in patients treated with best supportive care plus subcutaneous VA compared to best supportive care only. Results from our group could confirm these results in a health services research design [43]. In comparison to results of published survival analyses with standard care, median OS of stage IV NSCLC patients with resected brain metastases was 9.1 months (n = 64) [5]. In another study, shown by Sandler and collegues, OS of stage III/IV non-squamous NSCLC was 12.3 months after platinum-based doublets with bevacizumab as first- or second-line treatment [11]. In another study patients treated with docetaxel had an OS of 9.4 months, as shown by Borghaei and collegues [44]. Patients in the CTx group of the present study showed a median survival of 8 months which is comparable to the outcome of the CTx-arm in the Borghaei study. In the present study, patients receiving targeted therapy including bevacizumab or tyrosine kinase inhibitors and ICIs were not included as e.g. an imbalanced proportion of newer targeted or ICI treatment in one of the two comparing groups could have had an influential effect on the prolonging of survival in our study and would mask the impact of Ctx or VA treatment.

Our findings on overall survival are consistent with our results on PFS in that the patients treated with CTx + VA had a longer median PFS compared to the patients with CTx alone. However, the results of the PFS only showed a tendency towards significance, which is mainly owed to the fact that the time window for tumor recurrence is generally narrow in patients with stage IV NSCLC rather than to be explainable by patient number (80% of the total patient cohort were included in the PFS and RR analysis). Nevertheless, our findings of a significant improved partial tumor response in the combinational CTx + VA substantiate the improved overall survival of these patients. Hereby, longer add-on VA may additionally contribute to this outcome, being in line with results from a prospective randomized match-pair study [45].

Hazard analysis of the present study showed that male gender compared to female gender was significantly associated with an increased hazard of death being in line with previous studies [46–49]. Furthermore, the direction of survival impact of smoker status, malnutritional status and cancer-directed treatment in the present study and the significant positive association of hazard with the histology subtype large cell carcinoma are all well described in recently published data [49–53]. Interestingly, despite the fact that the authors of a randomized controlled trial showed that patients with adenocarcinoma had the greatest profit in terms of five-year survival with resected stage stage IB, II and IIIA NSCLC, they simultaneously concluded that the efficacy of adjuvant treatment was not dependent on histology type [54]. This is supported by another study in 2011 suggesting that histology subtype may not be predictive for outcome in advanced NSCLC treated with CTx [50]. It remains elusive and may be subject of future debates as to whether histology types serve as survival predictors.

With regards to their safety profile CTx-induced grade ≥3 toxicities including rash, anemia, diarrhea, and anorexia were found to be increased in combined CTx-targeted therapy compared to CTx [14, 55, 56] or compared to targeted monotherapy [14]. Despite the fact, that newer treatments such as ICIs are approved and on their way into guidelines some of them may bear safety gaps with respect to immune-related adverse events [57–60]. In addition, limited data exist to show their impact on HRQL. The rationale of applying add-on VA in oncological therapy is to improve HRQL of the patients and a Cochrane meta-analysis in 2008 acknowledged it’s positive impact on short-term HRQL of cancer patients during CTx [25]. A recently published integrative oncology guidelines marked VA therapy with evidence level C for improving HRQL [23, 24] in breast cancer patients. For many other cancer types a plethora of non-RCT type studies describe HRQL-improving effects of add-on VA (for a review see Kienle & Kiene [61, 62]. VA is also known to improve self-regulation in cancer patients [22, 25, 27, 63, 64]. Self-regulation is a “*problem solving capacity in terms of an active adaptation to stressful situations to restore well-being*” [65] and is regarded as the “*ability to actively achieve well-being*, *inner equilibrium*, *appropriate stimulation*, *a feeling of competence*, *and a sense of being able to control stressful situations*” [45]. The sound safety profile of add-on VA treatment [66, 67] or even the meliorating effect of VA on adverse events in combinational treatment with CTx [27, 28] or monoclonal antibody therapy is documented [32, 68]. Anti-proliferative and cytotoxic effects, latter due to pro-apoptotic (VA lectin) and pro-necrotic (VA viscotoxin) mode of actions in preclinical models have been described for VA extracts [69–72], for review see [73]. Recently, VA extracts were shown to inhibit proliferation and to bypass CTx-resistance of NSCLC cells in gene silencing *in vitro* strategies [74]. Furthermore, VA extracts possess antiangiogenic as well as anti-inflammatory and immunomodulatory properties *in vitro* [75–77] and *in vivo* [78] suggesting enhancement of humoral and cellular immune responses. Immunomodulatory mechanisms include among others IL-12 dependent activation of natural killer cells as shown by application of recombinant VA lectin in an animal model [79].

VA preparations contain several synergistically acting biologically active substances including e.g. lectins, viscotoxins or triterpene acids [80–82]. Generally, VA extracts are applied subcutaneously at low starting doses with a safe stepwise monitored dose adjustment depending on patient’s condition, tumor and immunological parameters [25]. In addition, intravenous and intratumoral applications of VA have been reported both being described as safe applications [66, 83]. Clinical outcome may depend on the composition of VA extracts, dose and length of application [45, 62]. Recent data indicate a survival benefit of VA or combinational CTx+VA-therapy in advanced or metastatic cancer patients [43, 84, 85]. In contrary, a prospective randomized phase II study of Bar-Sela and colleagues applying VA Iscador extracts in patients with NSCLC inaddition to platinum-based chemotherapy did not reveal survival improvement [27]. It has to be remarked, that survival was only the secondary endpoint of the Bar-Sela study and only half the patient number (n = 72) compared to our study has been enrolled. Even though Bar-Sela and colleagues did not find survival differences between treatment groups they observed a statistically significant increased CTx dose reduction (44%) in the control group (CTx only) compared to the add-on VA group (13%, p = 0.005) and the authors conclude that decreasing CTx dose reduction due to add-on VA may improve survival of these patients. In contrast, a meta analysis performed by Ostermann and collegues in 2009 [21] analyzing 41 eligible controlled clinical studies until 2008 on the clinical impact of adjuvant VA suggested its association with better survival of cancer patients (overall hazard ratio = 0.59 (CI: 0.53 to 0.66, p < 0.0001). A Cochrane report published in 2008 on VA therapy in oncology including randomized controlled trials analysed among other outcomes 13 eligible trials on survival in adults with any cancer type. Seven trials reported no, six trials reported a survival benefit. The authors concluded that VA had generally no consistent impact on disease free surival or overall survival. However, for lung cancer, the authors added that the evidence for non-superiority of VA is “limited to moderate” as only two trials were eligible. One trial included patients with inoperable lung cancer and one trial patients after surgery, both not with stage IV NSCLC.

Even though the evidence of VA’s impact on survival is discussed controversially [25–27], Ostermann and collegues summarized in their meta-analysis in 2009 that “*one can not ignore the fact that studies with positive effects of VA-E on survival of cancer patients are accumulating*” [21]. The results of our study fit into this statement and may, among other studies, be the basis for a prospective randomized controlled trial with combined CTx and VA in metastasized NSCLC.

Unwanted biases may have been introduced into the analysis, e.g. the assignment of treatment with add-on VA was performed in a non-randomized, non-controlled and un-blinded fashion and physicians could have unintentionally selected patients with better prognoses for VA therapy. Furthermore, it has been stated, that patients with a healthier lifestyle may be more open for additional integrative treatments and could have selected add-on VA therapy. As sound lifestyle data were not available, this aspect cannot be ruled out so far. Due to its specific mild to moderate local reactions such as erythema and flu-like symptoms it is difficult to apply VA in blinded studies which in most cases results in a lower grading in meta-analyses or reviews [86]. Further limitations of the present study may be its observational nature. Therefore, our findings and conclusions have to be handled with caution and should be interpreted in light of existing randomized, controlled trials. As suggested earlier, evidence for best treatment for patients “*should generally not be chosen based only on evidence from observational studies or single randomised clinical trials*” [87]. Even a circular model of evidence evaluation has been suggested by Walach and collegues, in which “*only a multiplicity of methods*, *which are used in a complementary fashion will eventually give a realitistic estimate of the effectiveness and safety of an intervention*”[88]. Therefore health service research data as presented in our RWD observational multicenter study may contribute to this and may complement the exisiting evidence of add-on VA therapy in oncological patients.

## Conclusions

Our findings suggest that patients with stage IV NSCLC receiving combined CTx plus VA therapy showed longest survival. The available data were of observational nature. Further prospective studies should focus on the effect of integrative treatment regimens including standard therapy and VA on survival and HRQL in more detail but would need to be planned in the light of emerging first- and second-line immuno- and combinational therapies.
