# Correction: Overall survival of stage IV non-small cell lung cancer patients treated with *Viscum album* L. in addition to chemotherapy, a real-world observational multicenter analysis

**DOI:** 10.1371/journal.pone.0273387

**Published:** 2022-08-16

**Authors:** Friedemann Schad, Anja Thronicke, Megan L. Steele, Antje Merkle, Burkhard Matthes, Christian Grah, Harald Matthes

The following information is missing from the Competing Interests statement:

Dr. Harald Matthes is a member of the board of directors at Weleda AG, a company that until 2016 distributed Iscador, a mistletoe preparation. In addition, Dr. Harald Matthes is a member of the board of the Hufelandgesellschaft e. V. in Germany; President of the DAHN (German Academy for Homeopathy and Naturopathy); and medical director and managing director of Gemeinschaftskrankenhaus Havelhöhe, a hospital for anthroposophic medicine. Dr. Harald Matthes is also a recipient on an endowed professorship from Charité in Berlin on Integrative and Anthroposophical Medicine. These competing interests do not alter the authors’ adherence to *PLOS ONE* policies on sharing data and materials.

## References

[1] Schad F, Thronicke A, Steele ML, Merkle A, Matthes B, Grah C, et al. (2018) Overall survival of stage IV non-small cell lung cancer patients treated with *Viscum album* L. in addition to chemotherapy, a real-world observational multicenter analysis. PLoS ONE 13(8): e0203058. 10.1371/journal.pone.0203058 30148853 PMC6110500

